# Knowledge, Perceptions, and Communication about Colorectal Cancer Screening among Chinese American Primary Care Physicians

**DOI:** 10.4137/CGast.S697

**Published:** 2008-05-16

**Authors:** Wenchi Liang, Mei-Yuh Chen, Grace X. Ma, Jeanne S. Mandelblatt

**Affiliations:** 1 Cancer Control Program, Lombardi Comprehensive Cancer Center, Georgetown University Medical Center, Washington, DC.; 2 Department of Public Health and Center for Asian Health, Temple University, Philadelphia, PA.

**Keywords:** cancer screening, communication, minority health, primary care

## Abstract

**Objective:**

To assess Chinese American primary care physicians’ knowledge, attitude, and barriers to recommending colorectal cancer (CRC) screening to their Chinese American patients.

**Methods:**

Chinese American primary care physicians serving Chinese American patients in two metropolitan areas were invited to complete a mailed survey on CRC screening knowledge, attitudes toward shared decision making and CRC screening, and CRC screening recommendation patterns.

**Results:**

About half of the 56 respondents did not know CRC incidence and mortality figures for Chinese Americans. Those aged 50 and younger, graduating from U.S. medical schools, or working in non-private settings had higher knowledge scores (*p* < 0.01). Physicians graduating from U.S. medical schools had more favorable attitudes toward shared decision making (*p* < 0.01). Lack of health insurance, inconsistent guidelines, and insufficient time were the most frequently cited barriers to recommending CRC screening.

**Conclusions:**

Most Chinese American physicians had knowledge, attitude, and communication barriers to making optimal CRC screening recommendations.

The Chinese are the largest sub-group of Asian Americans and Pacific Islanders (AAPI), the fastest growing and most culturally diverse minority population in the US.^1^ Colorectal cancer (CRC) is among the most commonly diagnosed cancers and the third leading cause of cancer death among Chinese Americans.^2, 3^ Although the Chinese have lower CRC incidence than Whites, when they do develop CRC, there are disparities in outcome, with the Chinese being diagnosed at later stages than other AAPI groups and Whites, suggesting missed screening opportunities.^4^

Chinese Americans, who are mostly foreign born,^1^ face unique cultural barriers to screening^5, 6^ and have among the lowest CRC screening rates in the US.^7^ Similar to what has been found in other groups,^8, 9^ physician recommendation is the most important determinant of CRC screening in older Chinese^10^ and has the potential to help Chinese patients overcome screening barriers. However, our previous research indicates that Chinese seeking care from Chinese physicians are only half as likely to receive physician recommendations or to have discussed CRC screening compared to those seen by English-speaking physicians.^11^

Little is known about the reasons for Chinese physicians’ low recommendation rates and there are no data on Chinese primary care physicians’ CRC knowledge, skills, or attitudes toward recommending CRC screening. To fill the knowledge gap, we conducted a survey of Chinese American primary care physicians practicing in the metropolitan Washington, D.C. and Philadelphia, P.A. areas to assess their knowledge, attitude, as well as cultural barriers to recommending CRC screening to their older Chinese American patients.

## Methods

### The study sample

Study protocols were approved by the Institutional Research Board of Georgetown University. Eligible physicians were those who: (1) were Chinese Americans, (2) practiced in the metropolitan Washington, D.C. or Philadelphia areas, (3) practiced primary care (in family medicine, general practice, internal medicine, or geriatrics), (4) had Chinese American patients aged 50 and older, and (5) could communicate with patients in Chinese (Mandarin, Cantonese, etc.)

The sample of 137 eligible physicians came from two sources. First, 74 eligible physicians were identified through Chinese American physician directories, Yellow Page advertisements, and our existing community networks. Next, we used a Chinese surname search of the American Medical Association (AMA) member database and further identified 63 physicians, after excluding duplicates and those not eligible or inactive (e.g. retired or moved).

### Data collection

The survey questionnaire, after being piloted on three Chinese American physicians, and the consent form were mailed to physicians in July and August 2006. Those not mailing or faxing the survey back in three weeks were followed up with a second mailing via registered mail with returned receipt. Those not returning the survey two weeks after the second mailing were followed up via phone for up to five times and provided the option of answering the survey over the phone or by an on-site interview. All respondents received a $50 gift card by mail.

### Measures

Background information included age, gender, place of birth, and place obtaining medical education. Physician practice characteristics included specialty, type and year of practice, and frequency of communicating with Chinese American patients in Chinese. Physicians’ knowledge about CRC and screening was assessed by seven statements about CRC incidence and mortality in Chinese Americans, CRC risk factors, and CRC screening guidelines (based on recommendations from the American Cancer Society [ACS] and U.S. Preventive Service Task Force).^12^ Physicians’ attitudes toward shared decision making (i.e. patients’ participation in the decision-making process) was assessed by 11 questions adapted from Liberati et al.^13^ Physicians’ attitudes towards discussing and recommending CRC screening were measured by 15 statements adapted from previous research,^14–16^ including perceptions about test benefits, barriers to recommendation, recommendation outcome, and cultural views. Finally, we assessed physicians CRC screening recommendation patterns by asking about the frequency, starting age, and intervals of recommendation and arrangements for their average-risk asymptomatic patients.

### Analysis

We first examined the frequency of responses and categorized them according to distribution. Knowledge was represented by number of correct answers to the seven questions. Attitudes toward shared decision making and CRC screening were calculated as sum scores of 11 and 15 items, respectively. Differences in knowledge, shared decision-making, and screening attitudes by physician background and practice patterns were analyzed using t-tests. The SAS version 9.1 (SAS, Inc., Cary, NC) was used to analyze the data.

## Results

### Knowledge, attitudes toward shared decision making

A total of 56 Chinese American physicians (40.9% of 137) completed the survey (Table 1). Twenty-nine (51.8%) and 24 (42.9%) of Chinese American physicians did not know the CRC incidence and mortality figures, respectively, among Chinese Americans. Those aged 50 and younger, having graduated from U.S. medical schools, or working in settings other than private practice had higher knowledge scores compared to those older than 50 years, graduated from non-U.S. medical schools, or in private practice (*p* < 0.01). Physicians receiving medical education in the U.S. had more favorable attitudes toward shared decision making (*p* < 0.01).

### Attitudes toward colorectal cancer screening

Lack of health insurance (85.2%), inconsistent guidelines (41.8%), and insufficient time (11.1%) were the most frequently cited barriers to recommending CRC screening (Table 2). Most Chinese physicians did not have a fatalistic view and would recommend CRC screening to their Chinese patients who emphasized self-care or had had negative impressions about medical examinations. Screening attitudes did not differ by physician characteristics. A higher CRC screening attitude score was significantly correlated with better knowledge and higher shared decision making scores (*p* < 0.01, data not shown).

### Colorectal cancer screening recommendation patterns

Physicians recommended CRC screening more often during routine health assessments than during other types of visits (Table 3). Twenty-nine (51.8%) and 19 (33.9%) of physicians did not recommend double contrast barium enema (DCBE) and flexible sigmoidoscopy (FS), respectively. About one-third started to recommend fecal occult blood tests (FOBT) before patients turned 50 years old. About half recommended colonoscopy every ten years and 29 (51.8%) reported prescribing home-based FOBT as suggested by the ACS guidelines.^12^

## Discussion

This is one of the first studies describing Chinese American primary care physicians’ knowledge and attitudes toward recommending CRC screening to their Chinese American patients. Most Chinese American physicians had knowledge, attitude, and communication barriers to making optimal CRC screening recommendations. Because Chinese Americans were more likely to seek care from Chinese American physicians, improving Chinese physicians’ knowledge and reducing recommendation barriers are important issues for improving Chinese American CRC screening rates.

Patterns of recommendations made by Chinese physicians in our study deviated from current guidelines,^12^ including not presenting all screening options and recommending tests at shorter intervals or starting at younger than recommended age. These findings are consistent with prior research in other primary care physician populations.^17,18^ Although screening guidelines recommend prepared, in-home FOBT, about half of the Chinese physicians in our study used a less accurate single-sample in-office test, which is higher than that (one third) reported in a national sample.^19^ Chinese physicians who were older, graduated from non-U.S. medical schools or in private practice were less knowledgeable of CRC and screening guidelines. This subgroup, which often treats a large pool of older Chinese American patients, is likely to benefit from educational and behavioral interventions designed to improve delivery of preventive cancer screening. Although our data do not have the power to detect the association between knowledge and recommendation patterns, future studies are needed to further examine the relationships using a larger sample of physicians.

Chinese physicians in our study did not share cultural views commonly seen in older Chinese American patients, such as fatalism and self-care.^5,20^ In fact, most of them did not identify cultural views of fatalism and self-care among their Chinese patients as barriers to CRC screening. In addition, they experienced recommendation barriers similar to those found in the general physician population, such as concerns about test sensitivity and specificity, lack of time to educate patients or perform tests, and the need to assign higher priority to patients’ other health concerns,^13–15^ and did not know the CRC risks specific to Chinese Americans. Our data suggest that Chinese American physicians have not been adequately trained to care for their Chinese American patients’ colorectal health, even though they have no difficulty in communicating in Chinese to those patients. Culturally sensitive communication skills are needed for these Chinese-speaking physicians. If properly trained, Chinese physicians are likely to better identify and address patient screening barriers than physicians who speak only English.

Several caveats should be considered when interpreting study results. First, the sample has limited power to detect difference between physician characteristics and recommendation patterns. Physicians from the mid-Atlantic region may not be representative of other Chinese American physicians practicing in other regions. Next, the screening recommendation patterns are obtained by self-report. Third, the fact that the survey was not completed anonymously may partly explain the moderate response rate. Also, it is possible that physicians not completing the survey were different from the participating physicians in certain aspects, which may influence the study results. For instance, if those lacking the knowledge of CRC and screening were more likely to decline, our current results may overestimate CRC knowledge among Chinese physicians. Fourth, we did not include a comparison group of non-Asian physicians; thus, no inference can be made about ethnic difference in physicians’ CRC screening recommendations.

In spite of the limitations, our results indicate that most Chinese American physicians did not have adequate knowledge of CRC risks in Chinese Americans. These physicians experienced recommendation barriers such as insufficient time for discussion and unfamiliarity with recommendation guidelines. This study provides important implications for future research about CRC screening recommendations among physicians serving minority populations like Chinese Americans. Chinese physicians may benefit from continuing education focusing on CRC knowledge specific to Chinese Americans, CRC screening guidelines supported by relevant research findings, and culturally appropriate training to identify and address Chinese Americans’ barriers, which, in turn, is likely to help increase CRC screening adherence in this underserved population.

## Figures and Tables

**Table 1.** Associations between physician characteristics and physician self-reported knowledge of colorectal cancer and screening and preferences for shared decision making (N = 56).

Physician characteristics		Knowledge of CRC and screening*	Shared decision making score^†^
Age			
	⩽50 years old (80.4%)	4.3^‡^	38.3
	>50 years old (19.6%)	2.6	36.2
Gender			
	Male (62.5%)	3.6	38.3
	Female (37.5%)	4.5	37.1
Birth place			
	US (32.1%)	4.2	39.2
	Asian country (67.9%)	3.8	37.3
Medical education			
	US medical school (55.4%)	4.7^‡^	39.6
	Non-US medical school (44.6%)	3.2	35.8
Year of practice			
	<10 years (53.6%)	4.2	38.0
	⩾10 years (46.4%)	3.6	37.8
Practice type			
	Private (60.7%)	3.5^‡^	37.2
	Other (39.3%)	4.7	39.0
Specialty			
	Internal medicine (64.3%)	3.6	38.4
	Other (35.7%)	4.5	36.9
Communicate with patients in Chinese			
	⩽50% of the time (42.9%)	3.8	38.3
	>50% of the time (57.1%)	4.1	37.6

* Number of correctly answered questions about knowledge of colorectal cancer and screening, ranging from 0 to 7.

† Sum score of 11 items, with higher numbers representing more positive attitudes toward shared decision making (range: 11 to 55).

‡ *p* < 0.01.

**Table 2.** Chinese American primary care physicians’ attitudes toward colorectal cancer and screening (N = 56).

Statement^a^		Strongly agree or agree (%)
**Benefits**		
1.	Screening for colorectal cancer is beneficial to my patients.	100
2.	Regular colorectal cancer screening can reduce colorectal cancer incidence and mortality.	92.6
3.	Screening for colorectal cancer is cost effective.	85.5

**Barriers**		
4.	Patients will not go for colorectal cancer screening if they do not have adequate health insurance coverage.	85.2
5.	Inconsistent recommendations about colorectal cancer screening make it difficult to decide which tests to offer.	41.8
6.	I do not have time to discuss or recommend colorectal cancer screening to patients coming in for other acute or chronic conditions.	16.7
7.	I do not have sufficient time to educate or motivate my patients to get colorectal cancer screening.	11.1
8.	Too many false positive or false negative results make me reluctant to recommend colorectal cancer screening to my patients.	10.9
9.	There are adequate laboratory and specialist resources (e.g. gastroenterology specialists) in my region for timely colorectal cancer screening referral.	81.8

**Recommendation outcome**		
10.	It is rewarding to see patients getting the colorectal cancer screening because of my recommendation.	87.3
11.	My patients will follow my recommendation to obtain colorectal cancer screening.	64.8

**Cultural views**		
12.	I do not feel the need to talk about colorectal cancer screening if my patients are healthy and take good care of them.	1.8
13.	I will not recommend colorectal cancer screening to patients who have negative impressions about medical examinations.	5.5
14.	Getting colorectal cancer is a matter of fate.	5.6
15.	No matter what I do, if a patient will get colorectal cancer, he or she will get it anyway.	7.3

a Each the statement was measured by a 5-point Likert scale, from “strongly agree,” “agree,” “neutral,” “disagree,” to “strongly disagree.”

**Table 3.** Patterns of colorectal cancer screening recommendations by Chinese American physicians (N = 56).

Patterns of CRC screening recommendation	FOBT	DCBE	FS	COL
Frequency				
During routine periodic health assessment				
Often	71.5%	0%	16.1%	87.5%
Sometimes	21.4%	39.3%	35.7%	12.5%
Rarely/Never	7.1%	60.7%	48.2%	0%
During other types of visits				
Often	19.7%	1.8%	5.4%	21.4%
Sometimes	57.1%	19.6%	35.7%	66.1%
Rarely/Never	23.2%	78.6%	58.9%	12.5%
Starting age				
<30 years old	1.8%	-	-	-
30~39 years old	3.6%	-	-	-
40~49 years old	23.2%	-	1.8%	-
50 years old (recommended)	66.1%	44.6%	60.7%	96.4%
55 years old	-	-	-	1.8%
60 years old	-	3.6%	1.8%	1.8%
Not applicable	-	51.8%	33.9%	-
Test interval, every				
½ year	1.8%	-	-	-
1 year (recommended for FOBT)	91.8%	-	-	-
2 years	3.6%	5.8%	1.9%	-
3–5 years	-	10.7%	42.9%	5%
5 years (recommended for DCBE, FS)	-	25%	21.4%	19.6%
5–9 years	-	-	-	23.2%
10 years (recommended for COL)	-	1.8%	-	50%
Not applicable	-	51.8%	33.9%	-
Test arrangements				
FOBT: Home-based test only	51.8%	N/A	N/A	N/A
DCBE: referred outside of practice (vs. within practice)	N/A	51.8%	N/A	N/A
FS: referred to others (vs. conducted by self)	N/A	N/A	66.1%	N/A
COL: referred outside of practice (vs. within practice)	N/A	N/A	N/A	91.1%

**Abbreviations:** CRC: Colorectal cancer; FOBT: Fecal occult blood test; DCBE: Double contrast barium enema; FS: Flexible sigmoidoscopy; COL: Colonoscopy.
